# The Short-Term Influence of Sibling Proactive and Reactive Aggression on Subsequent Peer Aggression

**DOI:** 10.3390/bs16081400

**Published:** 2026-08-15

**Authors:** Paula J. Fite, Selena A. Baca, Charlsie Solano, Emily Hichborn, Kat Wright, Annie L. Ryder

**Affiliations:** Department of Clinical Child Psychology, University of Kansas, Lawrence, KS 66045, USA; selenabaca@ku.edu (S.A.B.); charlsie.solano@ku.edu (C.S.); emily.g.hichborn@ku.edu (E.H.); katwright@ku.edu (K.W.); annie.ryder@ku.edu (A.L.R.)

**Keywords:** gender differences, proactive aggression, reactive aggression, sibling aggression

## Abstract

Although common, sibling aggression among youth is not well studied. This is a major omission in the literature, as sibling relationships have been found to influence other relationships. As such, the current study evaluates the links between self-reported sibling proactive and reactive aggression and peer proactive and reactive aggression six months later in a sample of 290 (54.8% male) elementary school-age youth who reported having at least one sibling. Gender differences in associations were also examined. Findings indicated that sibling reactive aggression was positively associated with peer reactive aggression six months later, over and above the stability of sibling proactive aggression and peer reactive aggression. Similarly, sibling proactive aggression was associated with peer proactive aggression over and above the stability of sibling reactive aggression and peer proactive aggression. However, gender differences were evident for proactive aggression. That is, sibling proactive aggression was associated with subsequent peer proactive aggression for boys only. Findings indicated that both sibling proactive and reactive aggression are associated with subsequent peer aggression within an academic year, suggesting the need to better understand sibling aggression for the prevention of aggression across relationships. Further, it appears that sibling proactive aggression is more influential on proactively aggressive behaviors toward peers for boys than girls.

## 1. Introduction

Sibling aggression is one of the most prevalent forms of interpersonal violence experienced during childhood (Renner et al., 2020). Based on United States and international samples, over a third of children and adolescents are perpetrators or victims of sibling aggression, which can include physical, psychological, or property destruction from one sibling to another (Finkelhor et al., 2015; Tippett & Wolke, 2015; Tucker et al., 2025; Qing et al., 2022). Importantly, perpetration and victimization of aggression are not mutually exclusive. Despite variability by context, sibling bullying (particularly in the form of aggression), is prevalent in notable portions of youth with siblings including those in the role of bullies (4–8%), victims (8–15%), and bully–victims (i.e., those who experience both perpetration and victimization; 13–29%) (Qing et al., 2022; Toseeb & Wolke, 2022). However, research examining sibling aggression has been limited relative to research on other forms of childhood aggression and other types of family or interpersonal violence (Caspi, 2012; Tompsett et al., 2018). Part of this pattern is the longstanding cultural tendency to view hostility between siblings as a normative, even developmentally inevitable, feature of growing up together (Caspi, 2012; Tucker et al., 2025). This paucity of research is particularly concerning, however, given that sibling aggression is linked to subsequent aggression towards others (Ensor et al., 2010; Natsuaki et al., 2009) and personal maladjustment (Tucker et al., 2015). One important next step in this research is examining how proactive and reactive functions of sibling aggression are related to subsequent peer aggression, as this research could inform aggression prevention and intervention approaches. Accordingly, the current study evaluated associations between sibling proactive and reactive aggression and peer proactive and reactive aggression 6 months later. Potential gender differences in associations were also evaluated.

### 1.1. Proactive and Reactive Functions of Aggression

Aggression is broadly defined as behavior that is intended to cause harm or pain towards another individual (Malti & Rubin, 2018) and is often categorized by its function, that is, why the individual engaged in the aggressive behavior (e.g., Fite et al., 2023). The two prominent aggression functions are proactive and reactive aggression. Proactive aggression represents aggressive acts that are used to obtain a desired outcome or goal (Card & Little, 2006; Dodge, 1991; Fite et al., 2023). For example, using physical force in order to dominate other kids. Individuals who engage in proactive aggression are more likely to demonstrate leadership qualities, be popular among their peers, and have a sense of humor (Dodge & Coie, 1987; Fite et al., 2023). Long-term consequences for youth who engage in proactive aggression include substance use, sex-, property-, and violence-related crimes (Miller & Lynam, 2006). In contrast, reactive aggression is represented by aggressive acts used in response to a perceived threat or provocation (Card & Little, 2006; Dodge, 1991; Fite et al., 2023). Individuals who engage in reactive aggression are more likely to experience peer rejection and display hostile attitudes, impulsivity, and challenges with maintaining their attention (Dodge et al., 1997; Fite et al., 2023). An example of reactive aggression would be striking back after being teased or threatened. Long-term consequences for youth who engage in reactive aggression include increased rates of delinquency, increased risk for domestic-related violence (Brendgen et al., 2001), and psychosocial maladjustments such as internalizing symptoms (Card & Little, 2006; Marsee & Frick, 2007).

Not only are proactive and reactive aggression differentially associated with maladjustment, but they also differ theoretically. Proactive aggression is best explained through the social learning theory (Bandura & Walters, 1977), which states that once aggression is learned, it is maintained through learning and reinforcement (Crick & Dodge, 1996). Theoretically, reactive aggression is best explained by the frustration-aggression hypothesis, which states that an individual engages in aggressive behavior as a means of defense when faced with perceived provocation (Bushman & Anderson, 2001; Pederson et al., 2023). Given these differing theoretical underpinnings and correlates of maladjustment, proactive and reactive aggressive behavior may not translate the same across contexts and relationships.

### 1.2. Sibling Aggression

A growing body of work positions sibling aggression as a form of family violence that is a serious public health concern (Tucker et al., 2025). Compared with peer aggression, sibling aggression is distinguished by the involuntary and enduring nature of the relationship and the privacy of the home setting in which it unfolds (Tucker et al., 2023). Further, research indicates that aggression toward siblings does not remain confined to the home. Youth often show patterns of continuity across settings, with elevated levels of aggression across both sibling and peer contexts (Tippett & Wolke, 2015). Longitudinal investigations have also demonstrated that children who behave aggressively toward a sibling are at elevated risk for engaging in later aggression with others, including peers (Ensor et al., 2010; Natsuaki et al., 2009).

Many factors contribute to the development and maintenance of aggression, including family dynamics and parenting approaches, social information processing, and environmental influences (e.g., neighborhood violence; e.g., Ostrov et al., 2018), and all of these factors may play a role in how sibling aggression contributes to subsequent peer aggression. Drawing on social learning theory, which posits that aggressive behaviors observed, modeled, and rehearsed within early family relationships are generalized to other interpersonal contexts (Bandura, 1973; Ingram et al., 2020). The sibling relationship may be especially well-suited to this rehearsal pattern since it is often enduring, emotionally charged, and, at times, unsupervised, providing repeated opportunities for aggressive strategies to be practiced, refined, and reinforced before being used elsewhere (Tucker et al., 2023). As such, aggressive behavior being modeled, practiced and reinforced at home may generalize to aggressive tendencies in other contexts, such as at school or in peer relationships.

Sibling aggression is also associated with broader behavioral and mental health concerns for aggressors. Given the maladaptive behavioral patterns reflected in aggressive tendencies, it is sensible that sibling aggression is associated with the development of externalizing presentations in youth (Toseeb & Wolke, 2022), as well as delinquent behavior more broadly (Tucker et al., 2015). The impact of sibling aggression on well-being is also observed beyond the effects of peer aggression (Tucker et al., 2013), such as significant links to anxiety and depression symptoms (Fite et al., 2021). As such, sibling aggression is not only important as it relates to childhood victimization, but it also reveals patterns of broader maladjustment and poor outcomes for the aggressor.

Sibling aggression, like peer aggression, can be differentiated by its underlying function. Extant research on sibling proactive and reactive aggression indicates that sibling proactive and reactive aggression are more stable than peer aggression and often occur at higher rates than peer aggression (Fite et al., 2021; Tucker et al., 2015). Further, the limited literature examining outcomes of proactive and reactive sibling aggression demonstrated similar associations with adjustment outcomes. Specifically, proactive sibling aggression has been linked to increased risk for substance use, whereas reactive sibling aggression is more closely associated with internalizing symptoms (Fite et al., 2021; Tucker et al., 2015).

Despite the importance of these functional distinctions, prior empirical work linking sibling aggression to subsequent peer aggression has largely focused on global indicators of aggression or victimization, missing the question of whether the functions of sibling aggression, proactive versus reactive, differentially predict peer aggression over time. Given evidence that proactive and reactive aggression have distinct pathways, social-cognitive correlates, and adjustment outcomes (Card & Little, 2006; Fite et al., 2023; Marsee & Frick, 2007), it is plausible that they exert differential influence on aggression directed at peers. Specifically, the emotional, impulsive nature of reactive aggression may translate across peer and sibling relationships. In contrast, individuals may learn that aggression is less effective for obtaining desired goals in peer relationships than in sibling relationships. In that case, the link between sibling proactive aggression and peer proactive aggression would be less strong than the link between sibling and peer reactive aggression.

### 1.3. Potential Gender Differences

Social learning theory (Bandura, 1973) posits that children learn aggression and other behaviors via interactions with others (e.g., siblings), and if this behavior is reinforced within one relationship or context is likely to be used as a strategy in other contexts and situations (e.g., peers). However, boys and girls are socialized differently and therefore may be differentially reinforced for the use of the various functions of aggression. As such, there may be gender-specific patterns in rates of proactive and reactive aggression as well as differential relations of proactive and reactive sibling aggression to peer aggression.

To date, the aggression literature is not consistent regarding gender differences in rates of proactive and reactive aggression. Specific to peer reactive aggression, some research suggests that boys display more reactive aggression (e.g., Baş & Yurdabakan, 2012), while other research indicates that boys and girls display equal levels of reactive aggression until children enter middle school (Guo et al., 2024). Findings for proactive aggression are equally mixed. In a clinically referred sample of youth, rates of proactive aggression were equal between boys and girls (Connor et al., 2003), while other school-based research has found that boys in late childhood are generally more proactively aggressive than girls (e.g., Baş & Yurdabakan, 2012; Guo et al., 2024). Even less is known about gender differences in sibling aggression. The limited cross-sectional (e.g., Solano et al., 2026) and longitudinal research (e.g., Fite et al., 2021) suggests no gender differences in functions of sibling aggression among middle- to late-childhood-aged youth. However, it is not clear whether links between sibling aggression and peer aggression differ across gender. Thus, further research evaluating whether gender differences exist is warranted, as this could impact whom to target for specific types of prevention and intervention approaches.

### 1.4. Current Study

Despite the prevalence of sibling and peer aggression (Renner et al., 2020; Ingram et al., 2020), as well as the known influence of sibling relationships on aggression against others (Ensor et al., 2010; Natsuaki et al., 2009), the influence of sibling proactive and reactive aggression on subsequent peer aggression has not been studied. The present study therefore sought to extend the sibling aggression literature by examining associations between proactive and reactive functions of sibling aggression and peer functions of aggression six months later, while controlling for prior levels of peer aggression. Further, given that previous findings regarding gender differences in proactive and reactive aggression are mixed and gender differences in sibling aggression are not well understood, potential gender differences were examined. It was expected that sibling proactive aggression would be positively associated with peer proactive aggression and that sibling reactive aggression would be positively associated with peer reactive aggression six months later. However, given the emotional and impulsive nature of reactive aggression (making this type of aggression less easy to control/inhibit), the association between sibling reactive aggression and subsequent peer reactive aggression was expected to be stronger than the link between sibling proactive aggression and subsequent peer proactive aggression. Further, given the strong associations between the two functions of aggression, it was anticipated that sibling reactive aggression may be associated with sibling proactive aggression and vice versa, but that these effects were not expected to be as strong as the associations with the same functions of aggression. With regard to potential gender differences, no specific hypotheses were posited given the mixed findings in the limited prior research.

Middle to late childhood is a developmental period during which peer relationships become increasingly centered around peer groups, particularly within the school setting (Shaffer & Kipp, 2013). During this stage, children develop social norms, learn expectations regarding cooperation and authority, and form more stable patterns of social and emotional functioning. Peer groups also become increasingly important for fostering belonging, social identity, and emotional support (Shaffer & Kipp, 2013). Research further suggests that peer proactive and reactive aggression increase as youth transition into early adolescence, particularly around the transition to sixth grade, before generally declining across adolescence (Fite et al., 2016). As social and emotional functioning becomes more established during this period, middle to late childhood represents an important developmental stage for examining proactive and reactive aggression, including how aggression towards siblings may influence aggression in peer relationships. Accordingly, the current study examined proposed associations in a sample of third through fifth grade youth.

## 2. Materials and Methods

### 2.1. Participants

Of the 422 third through fifth grade elementary students eligible to take part, 324 were enrolled in the study, yielding a participation rate of 76.77%. Among those 324 youth assessed at Time 1, 290 reported having one or more siblings and provided data on sibling aggression; this subset (54.8% boys) constituted the analytic sample. Participants were drawn from a Midwestern elementary school, where they were enrolled in Grades 3 through 5, and ranged from 8 to 11 years of age (M = 9.27, SD = 0.98). Children receiving special education services that would preclude their ability to fill out the survey independently were excluded. District-level demographic records indicated the following racial and ethnic composition of the student body: 85% non-Latinx White, 5% Black, 5% Latinx, 2% Asian, 2% Native American, and 1% multiracial (National Center for Education Statistics, 2019). The surrounding municipality reported a median household income of $32,338 (U.S. Census Bureau, 2019), and 36% of students within the district were eligible for free or reduced-price lunch (National Center for Education Statistics, 2019). At Time 2, data were collected from 266 of the original participants, reflecting a retention rate of 92%.

### 2.2. Measures

#### 2.2.1. Demographics

Participants self-reported their age and gender. Grade level was provided by the school administration.

#### 2.2.2. Proactive and Reactive Aggression

Sibling and peer proactive and reactive aggression were assessed using Dodge and Coie’s (1987) proactive and reactive aggression questionnaire, a six-item self-report measure that yields subscale scores for proactive (3 items; e.g., “I get other kids to gang up on someone that I do not like”) and reactive aggression (3 items; e.g., “When I have been teased or threatened, I get angry easily and strike back”). This measure was originally developed and validated as a teacher-report instrument (Dodge & Coie, 1987) but has since been widely adapted and validated for youth self-report with school-age children, including in studies examining sibling aggression specifically (Fite et al., 2009, 2021; Tampke et al., 2020). Notably, self-report versions of this measure have been used to directly compare proactive and reactive aggression toward siblings and peers within the same school-age samples (Fite et al., 2021), the same general approach used in the current study, and youth self-report on this measure has demonstrated expected patterns of construct validity, including associations with theoretically relevant correlates such as affective empathy (Tampke et al., 2020) and discrepancies from teacher report consistent with informant-specific perspectives rather than measurement error (Mildrum Chana et al., 2021). Items are ranked on a five-point Likert scale ranging from 1 (Never) to 5 (Almost Always). Mean scores for each subscale were computed for analysis, with higher scores reflecting higher levels of aggression. Participants completed two versions of the questionnaire—a peer-focused version and a sibling-version—to evaluate peer and sibling aggression separately. In the sibling-version of the questionnaire, the words “others,” “other children,” and “someone” were replaced with “my sibling” (e.g., “I feel that other children are to blame in a fight and feel that they started the trouble” was changed to “I feel that my sibling is to blame in a fight and feel that he or she started the trouble”). Youth were asked to complete these questions in relation to any of their siblings. Youth who did not have a sibling indicated “I don’t have a sibling” and were not included in the current study sample. Sibling aggression was measured at baseline (Time 1) and peer aggression was measured at baseline and at six-month follow-up (Time 2). Youth self-report on this measure has been found to yield borderline-to-adequate internal consistency across studies (α = 0.61–0.78), somewhat lower than parent- or teacher-report versions but consistent with expectations for a brief, three-item subscale format (Fite et al., 2024). In the current study, Cronbach’s alpha for sibling aggression indicated acceptable internal consistency for proactive aggression (α = 0.75) and good internal consistency for reactive aggression (α = 0.84); peer aggression alphas ranged from 0.64–0.69 for proactive aggression and 0.68–0.71 for reactive aggression. While some of these peer aggression alphas fell below the conventional threshold of 0.70 often cited as a minimum for acceptable reliability (Nunnally, 1978), values in this range are common for brief, three-item self-report subscales and are consistent with prior psychometric evaluations of this measure with youth informants (Fite et al., 2024).

### 2.3. Procedures

Data used in this study were collected in a rural Midwestern elementary school in the Fall of 2018 (Time 1) and again approximately six months later in the Spring of 2019 (Time 2). The six-month interval was chosen to allow sufficient time to observe behavioral changes in youth as social and emotional functioning continued to develop throughout the school year, while also limiting the administrative burden and interruption of scheduled curriculum caused by survey administration. This midterm measurement approach is common within school-based research (Salmela-Aro et al., 2021) and meets the minimum measurement requirement for cross-lagged panel designs (Hopwood et al., 2021). Study materials and procedures were approved by the researchers’ institutional review board and the school district administration prior to data collection. Information about the study and consent materials was provided to all parents of third-, fourth-, and fifth-graders as part of online back-to-school materials. After reviewing this information, parents completed consent procedures through the school’s online portal. Data were collected within individual classrooms during a single class period. At the beginning of the class period, youth whose parents did not provide consent were excused from the classroom. Study personnel then read an assent script aloud, describing the purpose and content of the survey, with youth following along with a written assent form. Youth indicated willingness to participate in the study by removing the assent form from their survey packet and holding it up for study personnel to collect. Youth who did not provide assent were excused from the classroom. No school personnel were present during data collection to encourage accurate responding. Youth completed a written survey while study personnel read survey items aloud to support reading comprehension and reduce the impact of lower reading ability. Research personnel also roamed the classroom to provide additional support to youth as needed. The survey took approximately 20 min to complete. Youth received pencils as compensation for their participation.

### 2.4. Data Analysis Plan

SPSS Version 29 statistical software (IBM Corp, 2022) was used to evaluate study objectives. Correlations were first examined to establish bivariate associations. Multiple regression analyses were then conducted to examine the associations between subtypes of sibling aggression and subtypes of peer aggression six months later, while controlling for initial levels of peer aggression, gender, and age. Interaction effects between sibling aggression and gender were then added to the models to determine whether these associations differed for boys versus girls. Note that all variables were standardized prior to regression analyses to reduce multicollinearity. Variance Inflation Factor (VIF) values were used to examine multicollinearity, with a conservative cutoff of <5.0 indicating multicollinearity is not a concern. Additionally, gender was coded as 0 (boys) and 1 (girls). Given that less than 10% of data were missing at Time 2, listwise deletion was utilized. This missing data approach resulted in sample sizes of 263 to 290 for correlation analyses and 260 to 266 for multiple regression analyses.

## 3. Results

### 3.1. Correlation Analyses

Correlations among study variables, along with means and standard deviations, are reported in Table 1. Sibling and peer aggression subtypes had moderate-to-strong positive associations with one another that were statistically significant (*p*s < 0.001), with one exception. Time 1 sibling reactive aggression was not associated with Time 1 peer proactive aggression (*p* = 0.291). Boys reported higher levels of Time 1 peer proactive aggression (*p* = 0.010) and higher levels of Time 2 peer reactive aggression (*p* = 0.029) than girls. No other significant gender differences (*p* = 0.076–0.861) or age differences (*p* = 0.072–0.732) were evident.

### 3.2. Longitudinal Links Between Sibling Aggression and Peer Aggression

Peer Reactive Aggression. Time 2 peer reactive aggression was simultaneously regressed on Time 1 sibling proactive and reactive aggression, Time 1 peer reactive aggression, age, and gender (see Table 2). VIF values ranged between 1.02 and 2.13. Both Time 1 sibling reactive aggression and Time 1 peer reactive aggression were positively associated with Time 2 peer reactive aggression, such that higher levels of reactive aggression were associated with higher levels of subsequent peer reactive aggression.

Peer Proactive Aggression. Time 2 peer proactive aggression was simultaneously regressed on Time 1 sibling proactive and reactive aggression, Time 1 peer proactive aggression, age, and gender (see Table 2). VIF values ranged between 1.02 and 1.92. Both Time 1 sibling proactive aggression and Time 1 peer proactive aggression were positively associated with Time 2 peer proactive aggression, such that higher levels of proactive aggression were associated with higher levels of subsequent peer proactive aggression. There was also a marginally statistically significant association between Time 1 sibling reactive aggression and Time 2 peer proactive aggression (*p* = 0.065), such that higher levels of Time 1 sibling reactive aggression were associated with higher levels of Time 2 peer proactive aggression.

### 3.3. Gender Differences

Peer Reactive Aggression. When gender was examined as a moderator of the association between Time 1 sibling reactive aggression and Time 2 peer reactive aggression, no statistically significant interaction effect was evident (β = −0.05, 95% CI [−0.14, 0.05], SE = 0.05, *p* = 0.308).

**Figure 1:**
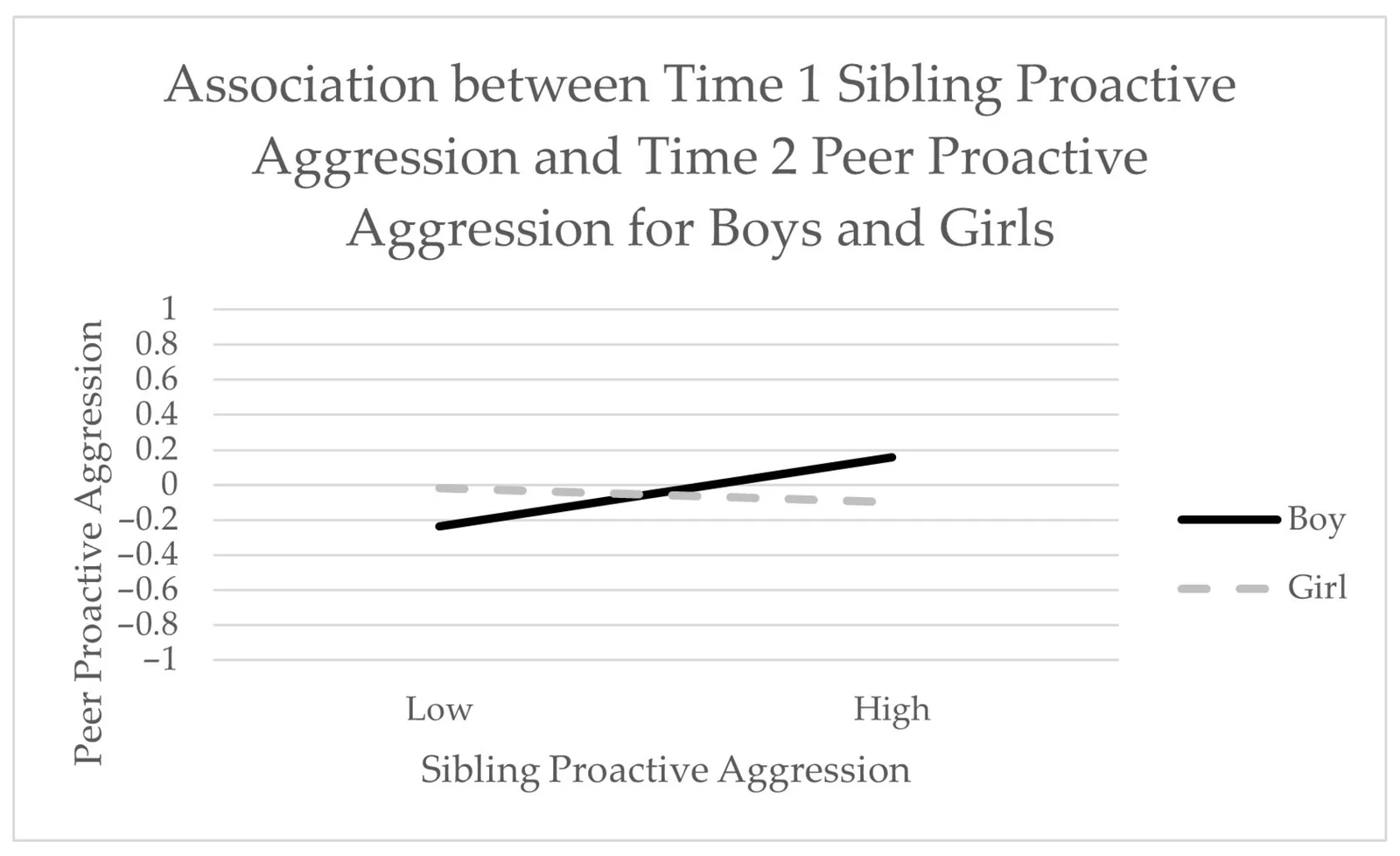
Association between Time 1 Sibling Proactive Aggression and Time 2 Peer Proactive Aggression for Boys and Girls.

Peer Proactive Aggression. When gender was examined as a moderator of the association between Time 1 sibling proactive aggression and Time 2 peer proactive aggression, a statistically significant interaction effect was evident (β = −0.12, 95% CI [−0.23, −0.01], SE = 0.06, *p* = 0.036). As shown in Figure 1, simple slope analyses indicated that for boys, higher levels of sibling proactive aggression were associated with higher levels of Time 2 peer proactive aggression (β = 0.20, 95% CI [0.06, 0.34], SE = 0.07, *p* = 0.005). However, the association between Time 1 sibling proactive aggression and Time 2 peer proactive aggression was not statistically significant for girls (β = −0.04, 95% CI [−0.26, 0.18], SE = 0.11, *p* = 0.715).

## 4. Discussion

The current study extends prior aggression research by examining the short-term influence of sibling aggression on subsequent peer aggression while considering prior levels of peer aggression. More specifically, associations between sibling and peer proactive and reactive aggression were evaluated, and potential gender differences in these links were examined. The results support that sibling aggression represents a distinct class of aggressive behavior with relevant predictive value for understanding subsequent aggression across contexts, particularly among peers.

As hypothesized, both functions of sibling aggression were specifically linked to peer functions of aggression. This is consistent with prior research supporting that sibling aggression is associated with subsequent aggression in other relationships (e.g., Ensor et al., 2010; Natsuaki et al., 2009). However, the current study further advances prior work by evaluating the links between specific functions of sibling aggression to peer aggression and further indicates that the link between sibling proactive aggression and subsequent peer proactive aggression was unique to boys in this sample. This is consistent with cross-cultural research suggesting that males are more likely than females to engage in physical aggression in nonfamilial contexts (Varnum et al., 2025). Perhaps for boys, proactive aggression is more socially acceptable, and therefore the experience of engaging in proactive aggression towards siblings is more strongly linked to engaging in such behavior with peers (e.g., Varnum et al., 2025). For example, research has found that parents are more willing to tolerate aggressive acts from boys than girls (Martin & Ross, 2005). Additionally, there could also be hormonal differences, particularly testosterone, that influence the use of aggression in various relationships (e.g., Book et al., 2001). However, more research is needed to evaluate and fully understand this gender-specific link, particularly with regard to proactive and reactive functions of aggression.

It was posited that sibling reactive aggression may be more strongly linked to peer reactive aggression than sibling proactive aggression to peer proactive aggression due to the emotionful and impulsive nature of reactive aggression (e.g., Brendgen et al., 2001; Fite et al., 2023). However, this pattern was not observed. It may be that youth are skilled in translating and adapting their planful aggression toward their siblings to peers, where they continue to use aggression to help them obtain their desired goals with their peers (Fite et al., 2023). Prior research supports that sibling aggression is associated with aggression across relationships (Ensor et al., 2010; Natsuaki et al., 2009), and this appears to be similar for both proactive and reactive aggression in this age group.

Further, there was a marginally statistically significant trend for sibling reactive aggression to be uniquely associated with peer proactive aggression. Reactive aggression is the more common function of aggression, and prior research suggests it often precedes proactive aggression (for reviews see: Fite et al., 2023; Vitaro & Brendgen, 2011). Thus, this link could reflect a developmental trend or cascade of aggressive behavior from reactive to proactive aggression in addition to sibling aggression being associated with peer aggression.

The influence of sibling aggression on peer aggression was above and beyond prior levels of aggression, as both peer proactive and reactive aggression were stable over the six months. These findings are consistent with prior research indicating stability in proactive and reactive aggression (Fite et al., 2023; Vitaro & Brendgen, 2011), and current findings further highlight the impact of sibling aggression on subsequent aggression in other relationships, specifically peer relationships. Consistent with social learning theory, that behavior is often the result of observing, modeling, and reinforcement (Bandura, 1973; Ingram et al., 2020), family dynamics (particularly siblings based on the current findings) appear to shape our future interactions and relationships with others. Other factors within one’s environment, including parenting behaviors and community aggression/violence, also contribute to sibling aggression as well as subsequent aggression in nonfamilial relationships (Ostrov et al., 2018).

While findings from the present study increase our understanding of the developmental trajectories of aggression in school-age children, several limitations should be considered. First, this sample consisted of primarily White, English-speaking youth in a rural community. Additionally, information on socioeconomic standing, race, or ethnicity was not collected at the individual level. As such, group-level differences in sibling and peer aggression could not be evaluated, and findings may not generalize to more diverse or urban populations. Data were also only collected from students in third through fifth grades, and findings may not be generalizable to older or younger youth. Research in more diverse populations is needed to understand the generalizability of these findings. Further, only self-report data were collected, which may be subject to social desirability, recall, and response biases, as well as common method variance. Additionally, some aggression internal consistencies, particularly for peer aggression, fell below conventional thresholds, which is likely attributable to the brevity of the subscales (three items each) and is consistent with prior work indicating that youth self-report on this measure tends to show lower, though still acceptable, reliability relative to parent- or teacher-report versions (Fite et al., 2024). This modest reliability may have attenuated observed associations, suggesting that true effect sizes could be somewhat larger than reported here; findings should be interpreted with this measurement limitation in mind. Future studies would benefit from the inclusion of caregiver and teacher report, as well as observational measures, to confirm findings. Finally, variables were assessed over a six-month timeframe. While the longitudinal design of this study is a strength, additional research is needed to evaluate these associations over a longer timeframe to better understand their stability and developmental trajectory.

Despite these limitations, the study findings emphasize the need to ensure that sibling aggression is not dismissed as normative behavior. Sibling aggression represents a significant risk factor for aggressive tendencies outside of the home, particularly aggression toward peers, and should be addressed early. This work informs the development of early intervention approaches for childhood aggression by supporting the need to target sibling aggression as an avenue for mitigating later patterns of peer aggression. This suggests that parental psychoeducation regarding sibling aggression is warranted. The results also add to the literature informing gender differences in developmental patterns of aggression. Specifically, the results reveal how cross-context pathways can differ by gender, particularly the added risk observed in pathways involving sibling and peer proactive aggression. Current intervention recommendations include culturally attuned family-based approaches that consider gender and family dynamics in how to best mitigate aggression, which can include strategies such as conflict resolution and not showing favoritism (Edet, 2025).

Future research should examine if cross-context links also vary by age, specifically among adolescents. Adolescence often reflects a period of increased autonomy, changing sibling dynamics, and more complex peer relationships (Allen & Loeb, 2015; McHale et al., 2012). Therefore, patterns may differ for older youth. Additionally, sibling-related covariates, including number of siblings, birth order, and age gap, all of which have been shown to influence sibling relations (Jensen et al., 2024; Van et al., 2017), were not collected in the current study and should be included in future research. Next steps in this work should also incorporate the examination of forms of aggressive behavior. Understanding risk patterns by function (i.e., the reason/purpose of the behavior for the aggressor), as in the current study, is valuable to understand why youth may or may not engage in certain types of behavior. The incorporation of specific forms of aggressive sibling behavior that are linked to peer aggression could provide insight into how presentations that reflect risk may look (e.g., if children are aggressive toward siblings in a physical vs. relational manner, or overt vs. covert manner). Exploring other potential moderators that play a role in these pathways, such as consequences/discipline for aggressive behavior in the home, can also inform patterns of risk. Such examination would add further dimension to the present findings that sibling aggression contributes to the development of peer aggression during middle to late childhood.

## 5. Conclusions

The current study contributes to the aggression literature by further evaluating the links between sibling proactive and reactive and aggression and peer aggression 6 months later in middle to late childhood. Results indicate that sibling reactive aggression is associated with subsequent peer reactive aggression for both boys and girls. However, sibling proactive aggression is only associated with peer proactive aggression among boys. Findings emphasize the need to address sibling aggression for the prevention of subsequent peer aggression and further suggest that gender specific interventions may be indicated.

## Figures and Tables

**Table 1:** Correlations among Study Variables.

	1	2	3	4	5	6	7	8
1. Age	-							
2. Gender	−0.10	-						
3. T1 Sibling Reactive	0.02	0.01	-					
4. T1 Sibling Proactive	−0.08	−0.05	0.63 **	-				
5. T1 Peer Reactive	0.07	−0.10	0.38 **	0.45 **	-			
6. T1 Peer Proactive	−0.03	−0.15 *	0.06	0.32 **	0.55 **	-		
7. T2 Peer Reactive	0.11	−0.13 *	0.46 **	0.40 **	0.58 **	0.21 **	-	
8. T2 Peer Proactive	0.03	−0.09	0.23 **	0.40 **	0.58 **	0.50 **	0.59 **	-
Mean	9.27	-	1.87	1.25	1.58	1.12	1.65	1.11
Standard Deviation	0.98	-	1.06	0.65	0.78	0.42	0.75	0.37

Note. T1 = Time 1; T2 = Time 2; * *p* < 0.05, ** *p* < 0.001.

**Table 2:** Multiple Regression Analyses.

	T2 Peer Reactive Aggression				T2 Peer Proactive Aggression			
	β	[95% CI]	SE	*p*	β	[95% CI]	SE	*p*
Age	0.06	[−0.04, 0.15]	0.05	0.256	0.03	[−0.06, 0.12]	0.04	0.475
Gender	−0.08	[−0.17, 0.02]	0.05	0.104	−0.002	[−0.09, 0.09]	0.04	0.969
T1 Sibling Reactive Aggression	0.27 *	[0.15, 0.40]	0.06	<0.001	0.11 ^†^	[−0.01, 0.22]	0.06	0.065
T1 Sibling Proactive Aggression	−0.03	[−0.16, 0.11]	0.07	0.668	0.15 *	[0.02, 0.28]	0.07	0.025
T1 Peer Aggression	0.43 *	[0.31, 0.55]	0.06	<0.001	0.24 *	[0.13, 0.35]	0.06	<0.001

Note. T1 Peer aggression represents reactive aggression for the T2 peer reactive aggression models and proactive aggression for the T2 peer proactive aggression model; * *p* < 0.05, ^†^ *p* < 0.07.

## Data Availability

Data are available upon request from the first author.
